# Indoxyl sulfate potentiates skeletal muscle atrophy by inducing the oxidative stress-mediated expression of myostatin and atrogin-1

**DOI:** 10.1038/srep32084

**Published:** 2016-08-23

**Authors:** Yuki Enoki, Hiroshi Watanabe, Riho Arake, Ryusei Sugimoto, Tadashi Imafuku, Yuna Tominaga, Yu Ishima, Shunsuke Kotani, Makoto Nakajima, Motoko Tanaka, Kazutaka Matsushita, Masafumi Fukagawa, Masaki Otagiri, Toru Maruyama

**Affiliations:** 1 Department of Biopharmaceutics, Graduate School of Pharmaceutical Sciences, Kumamoto University, Japan; 2 Center for Clinical Pharmaceutical Sciences, School of Pharmacy, Kumamoto University, Kumamoto, Japan; 3 Department of Organic Chemistry, Graduate School of Pharmaceutical Sciences, Kumamoto University, Kumamoto, Japan; 4 Department of Nephrology, Akebono Clinic, Kumamoto, Japan; 5 Division of Nephrology, Endocrinology and Metabolism, Tokai University School of Medicine, Kanagawa, Japan; 6 Faculty of Pharmaceutical Sciences, Kumamoto, Japan; 7 DDS Research Institute, Sojo University, Kumamoto, Japan

## Abstract

Skeletal muscle atrophy, referred to as sarcopenia, is often observed in chronic kidney disease (CKD) patients, especially in patients who are undergoing hemodialysis. The purpose of this study was to determine whether uremic toxins are involved in CKD-related skeletal muscle atrophy. Among six protein-bound uremic toxins, indole containing compounds, indoxyl sulfate (IS) significantly inhibited proliferation and myotube formation in C2C12 myoblast cells. IS increased the factors related to skeletal muscle breakdown, such as reactive oxygen species (ROS) and inflammatory cytokines (TNF-α, IL-6 and TGF-β1) in C2C12 cells. IS also enhanced the production of muscle atrophy-related genes, myostatin and atrogin-1. These effects induced by IS were suppressed in the presence of an antioxidant or inhibitors of the organic anion transporter and aryl hydrocarbon receptor. The administered IS was distributed to skeletal muscle and induced superoxide production in half-nephrectomized (1/2 Nx) mice. The chronic administration of IS significantly reduced the body weights accompanied by skeletal muscle weight loss. Similar to the *in vitro* data, IS induced the expression of myostatin and atrogin-1 in addition to increasing the production of inflammatory cytokines by enhancing oxidative stress in skeletal muscle. These data suggest that IS has the potential to accelerate skeletal muscle atrophy by inducing oxidative stress-mediated myostatin and atrogin-1 expression.

Skeletal muscle atrophy, referred to as sarcopenia, is often observed in chronic kidney disease (CKD), especially in patients who are undergoing hemodialysis, and is correlated with the risk of morbidity and mortality in such patients^1^,^2^. In catabolic conditions such as CKD, persistent imbalances between protein synthesis and degradation cause a substantial loss of muscular protein in which the ubiquitin-proteasome system (UPS) and myostatin have emerged as functioning as inducers of muscle wasting^3^,^4^. Bodine *et al*. reported that the expression of atrogin-1, a member of muscle specific ubiquitin ligase family, was increased under conditions of skeletal muscle atrophy and mice lacking atrogin-1 were resistant to muscle atrophy^5^. These findings indicate that both myostatin and atrogin-1 play an important role in skeletal muscle atrophy^6^.

Myostatin, a member of the transforming growth factor-β family is produced primarily in skeletal muscle, and functions as a negative regulator of muscle growth^7^. Recently, Zhang *et al*. reported that myostatin expression in muscle was increased in patients with CKD^8^ as well as five-sixth nephrectomized mice (CKD mice), and that the administration of anti-myostatin anti-peptide to these mice suppressed the reducing of muscle mass^9^,^10^. They also found that inflammatory cytokines such as TNF-α, IL-6 and TGF-β1 levels, which were known to cause skeletal muscle break down, were also increased in muscles of CKD mice, whereas the inhibition of myostatin reduced the levels of these cytokines in the circulation^9^. Moreover, Cheung *et al*. demonstrated that the infusion of cytokines such as TNF-α and IL-6 into mice results in the development of muscle atrophy, while it was attenuated by the neutralization of these cytokines^11^. Zhang *et al*. also reported that TNF-α activates myostatin, which further accelerates UPS-mediated catabolism^9^. Similar to myostatin, atrogin-1 was also regulated by oxidative stress and inflammatory cytokines. These findings point to the conclusion that the development of skeletal muscle atrophy is mutually linked with myostatin, atrogin-1, inflammation and oxidative stress^12^,^13^,^14^,^15^,^16^,^17^. However, the initiating factors responsible for the onset and progression of skeletal muscle atrophy in CKD remains poorly understood.

Uremic toxins are accumulated in the body under CKD conditions and are considered to exert detrimental actions. Among these toxins, the presence of protein-bound uremic toxins such as indoxyl sulfate (IS), indole acetic acid (IA), *p*-cresyl sulfate (PCS), hippuric acid (HA), kynurenic acid (KA) and 3-carboxy-4-methyl-5-propyl-2-furanpropanoic acid (CMPF) has been noted, due to the difficulty of removing them by hemodialysis because of their strong binding affinity for serum albumin^18^. Recent accumulating evidence has clarified that protein-bound uremic toxins are related to renal toxicity and CKD complications, including cardiovascular damage caused by enhanced oxidative stress and inflammation^19^,^20^. Several mechanisms have been proposed to explain the detrimental actions of uremic toxins. For instance, uremic toxins enter the target cell *via* specific transporters, such as an organic anion transporter (OAT)^21^,^22^,^23^, and then, they exert their toxicity *via* the activation of cellular NADPH oxidase, which results in the overproduction of reactive oxygen species (ROS) and inflammatory cytokines^19^,^20^. In addition, recent reports have shown that indole contained toxins, especially IS, acts as an aryl hydrocarbon receptor (AHR) ligand and exerts its toxicity *via* AHR^24^,^25^. Interestingly, Ohake *et al*. reported that AHR functions as a component of the ubiquitin ligase complex^26^. Since oxidative stress and inflammation play an important role in the development of skeletal muscle atrophy, as mentioned above^14^,^15^,^16^,^17^, we hypothesized that uremic toxins, especially, a substrate of AHR, can trigger muscle atrophy by increasing oxidative stress and inflammation *via* NADPH oxidase or the AHR pathway. However evidence regarding an association between uremic toxins and skeletal muscle atrophy is not currently available. Understanding the mechanism by which uremic toxins regulate muscle mass promise to provide new therapeutic targets for the prevention and treatment of muscle atrophy in patients with CKD.

In the present study, we examined the involvement of protein-bound uremic toxins on skeletal muscle atrophy. We first tested the *in vitro* effects of six protein-bound uremic toxins on cell proliferation and differentiation (myotube formation), or oxidative stress using a mouse myoblast cell line (C2C12), and its effects on inflammation, myostatin expression, muscle atrophy- or myogenic-related genes expression and Akt phosphorylation in C2C12 cells are also then evaluated. Finally, to determine if the pathway obtained from an *in vitro* study, was operative *in vivo*, we evaluated the contribution of IS to the development of muscle atrophy using IS-overloaded half-nephrectomized mice. The findings reported here indicate that IS is a potent uremic toxin that accelerates the development of skeletal muscle atrophy associated with CKD by inducing the oxidative stress-mediated expression of myostatin and atrogin-1.

## Results

### The effect of uremic toxins on the proliferation and myotube formation of C2C12 myoblast cells

**Figure 1:**
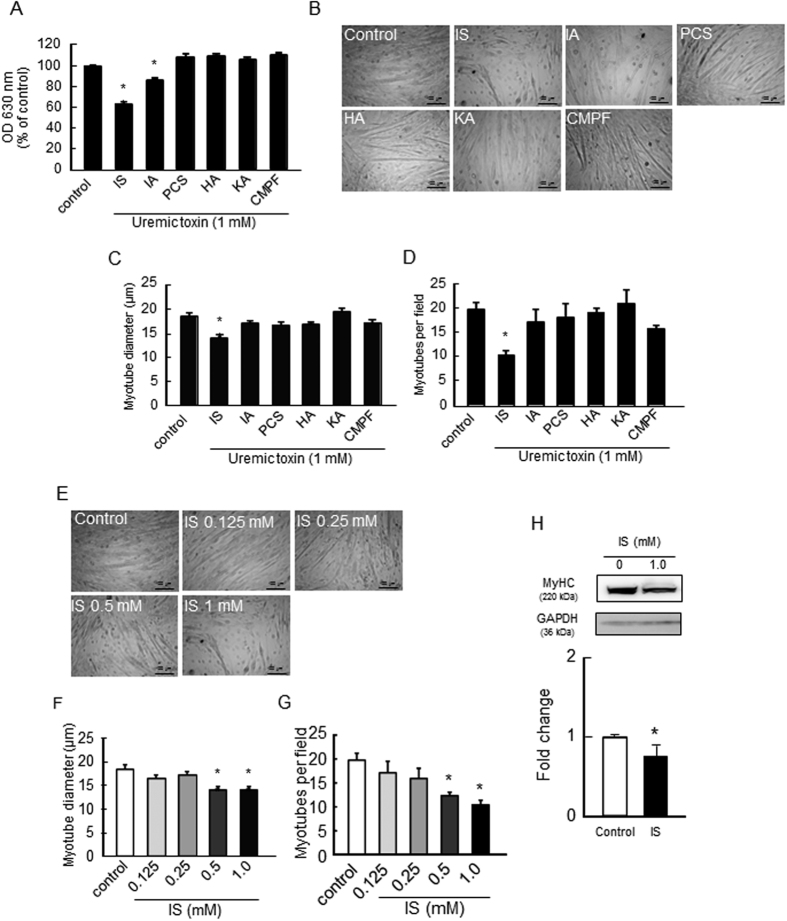
Effect of uremic toxins on the proliferation and myotube formation of C2C12 myoblast cells. (**A**) Effect of uremic toxins on C2C12 myoblast cell proliferation was determined by a methylene blue assay. C2C12 myoblast cells were seeded on a 96-well plate and cultured overnight. After adhesion, cells were treated with uremic toxins (1 mM) and incubated for 72 hr. Adherent cells were fixed and stained with methylene blue. The OD630 nm was then measured. Effect of uremic toxins on cell differentiation was determined by C2C12 myoblast cell tubular formation. C2C12 myoblast cells were seeded on a 12-well plate and cultured overnight. After cell adhesion, cultured medium was changed to differentiation medium containing with uremic toxins and cultured for 7 days. The cell density in each treatment group was the same before changing the culture medium to differentiation medium. (**B**) Cell morphology was observed by microscopy, and (**C**) average diameters of at least 50 myotubes were determined to be 40 μm along the length of the myotube. (**D**) The number of the myotubes per field was measured 5 fields per sample. Myotubes were counted, and diameter was assessed on Image J. Dose-dependent effect of IS on (**E**) cell morphology, (**F**) myotube diameter, and (**G**) the number of myotubes were shown. Scale bar = 100 μm. (**H**) Myotube formation was also evaluated using second marker, namely, the myosin heavy chain (MyHC) by Western blot. Data are expressed the means ± SEM (n = 3~6). *p < 0.05, **p < 0.01 compared with control.

We first examined the effect of uremic toxins on cell proliferation and myotube formation of C2C12 myoblast cells. Among the uremic toxins, we focused on six protein-bound solutes, namely, IS, IA, PCS, HA, KA and CMPF due to their high accumulation under CKD conditions and their detrimental effects. The antiproliferative effect of these uremic toxins (1 mM) was examined at 72 hr in C2C12 myoblast cells that were treated with these solutes. As a result, IS showed a strongest antiproliferative effect (Fig. 1A). Similarly, IA also significantly inhibited cell proliferation, but to a lesser extent. Since C2C12 myoblast cells form a myotubular morphology through differentiation^27^, myogenic differentiation was induced by changing the incubation media from 10% fetal bovine serum to 2% horse serum. Continuous treatment with IS reduced myotube diameter by 24% and the number of myotubes by 50% during 7 days (Fig. 1B–D) in a dose-dependent manner (Fig. 1E–G). Myotube formation was also evaluated using a second marker, the myosin heavy chain by Western blot. As a result, the IS treatment also decreased the expression of the myosin heavy chain (Fig. 1H). In contrast, the other toxins had negligible effects on myotube formation of C2C12 myoblast cells.

### Effect of uremic toxins on ROS production in C2C12 myoblast cells

**Figure 2:**
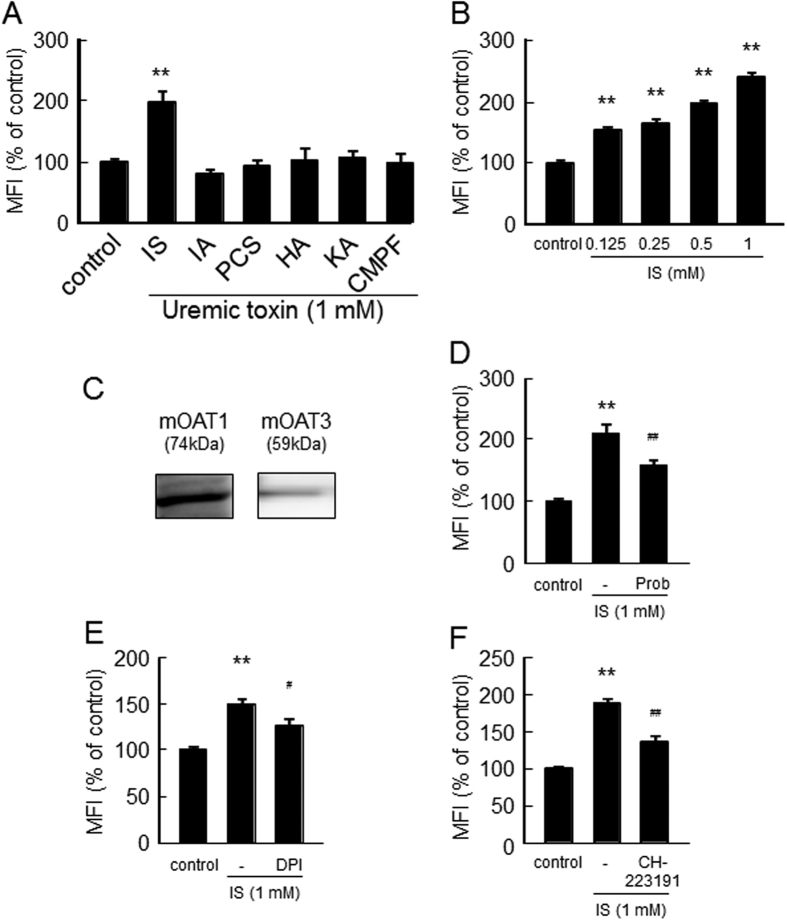
Effect of uremic toxins on ROS production in C2C12 myoblast cells. (**A**) Effect of uremic toxins on ROS production was determined using CM-H_2_DCFDA. C2C12 myoblast cells were starved with serum free medium for 2 hr, and then treated with CM-H_2_DCFDA in D-PBS for 30 min. After removal of the D-PBS, the cells were treated with uremic toxins and incubated for 2 hr. Fluorescence intensity was measured at an excitation wavelength of 485 nm and at an emission wavelength of 535 nm. (**B**) Dose-dependent effect of IS on ROS production was measured in C2C12 myoblast cells (**C**) Expression of mouse Oat1 and Oat3 in C2C12 cells was detected by Western blotting. To determine the effect of inhibitors of Oats, NADPH oxidase or AHR on the IS-induced ROS production, cells were incubated with CM-H_2_DCFDA for 30 min followed by incubation with (**D**) Oats inhibitors (probenecid (0.5 mM)), (**E**) NADPH oxidase inhibitor (diphenylene iodonium: DPI (50 μM)) or (**F**) AHR inhibitor (CH223191 (10 μM)) for 30 min. The cells were then incubated with IS (1 mM) for 1 hr with Oats inhibitors or 2 hr with others. Data are expressed the means ± SEM (n = 6). **p < 0.01 compared with control. ^#^*p* < 0.05, ^##^*p* < 0.01 compared with IS only.

Since ROS plays an important role for skeletal muscle atrophy^14^,^15^,^16^,^17^, we examined the effect of six uremic toxins on ROS production in C2C12 myoblast cells. As shown in Fig. 2A, 1 mM IS significantly increased intracellular ROS levels in C2C12 myoblast cells. Among them, IS showed the strongest ROS production in a dose-dependent manner and even at IS concentration of 125 µM (Fig. 2B). These results are consistent with the antiproliferative effect or reducing myotube formation of IS, as observed in Fig. 1.

To explore the mechanism responsible for the pro-oxidant properties of IS in muscle cells, we examine the effect of various inhibitors on the IS-induced ROS production. Mouse Oat1 and Oat3 were expressed by C2C12 myoblast cells (Fig. 2C). As shown in Fig. 2D, probenecid, an Oat inhibitor, significantly inhibited IS-induced ROS production. Similar inhibitions were also observed in the presence of an NADPH oxidase inhibitor (DPI) and an AHR inhibitor (CH-22319)(Fig. 2E,F). These data suggest that the intracellular accumulation of IS followed by the activation of NADPH oxidase and the AHR pathway are involved in the process of ROS generation in C2C12 myoblast cells.

### Effect of IS on the expression of inflammatory cytokines in C2C12 myoblast cells

**Figure 3:**
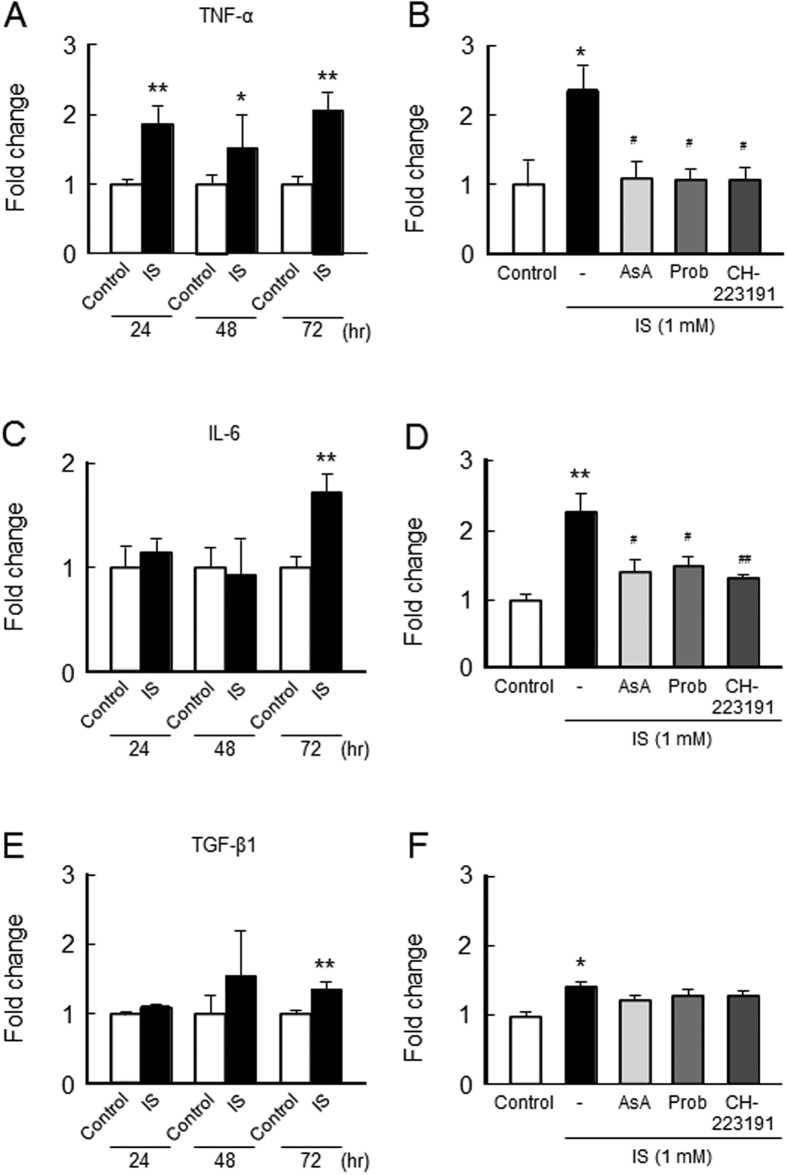
Effect of IS on inflammatory cytokine expression on C2C12 myoblast cells. Effect of IS on inflammatory cytokines expression was determined by real time RT-PCR. C2C12 myoblast cells were starved with serum free medium for 2 hr, and then treated with IS for 24, 48 or 72 hr. After the incubation, total RNA was collected and the mRNA expression of (**A**) TNF-α, (**C**) IL-6 or (**E**) TGF-β1 in C2C12 myoblast cells were determined. To determine the effect of inhibitors, cells were co-incubated in the presence or absence of ascorbic acid (AsA, a ROS scavenger), probenecid (Prob, Oat inhibitor) and CH-223191 (AHR inhibitor) for 72 hr, and the mRNA expression of (**B**) TNF-α (**D**) IL-6 or (**F**) TGF-β1 in C2C12 myoblast cells were determined. Data are expressed the means ± SEM (n = 3~5). *p < 0.05, **p < 0.01 compared with control. ^#^*p* < 0.05, ^##^*p* < 0.01 compared with IS only.

We next examined the issue of whether IS-induced ROS production results in an increase in inflammatory cytokines (TNF-α, IL-6 and TGF-β1) expression. As shown in Fig. 3A, the mRNA expression of TNF-α began to increase at 24 hr after the IS treatment and this was maintained for a period of up to 72 hr. On the other hand, the mRNA expression of IL-6 or TGF-β1 increased at 72 hr after the IS treatment (Fig. 3C,E). Interestingly, ascorbic acid, a ROS scavenger, probenecid and CH-223191 significantly inhibited IS-induced TNF-α and IL-6 expression, while they tended to suppress TGF-β1 expression (Fig. 3B,D,F).

### Effect of IS on the expression of myostatin, skeletal muscle atrophy- or myogenic-related genes and Akt phosphorylation in C2C12 myoblast cells

**Figure 4:**
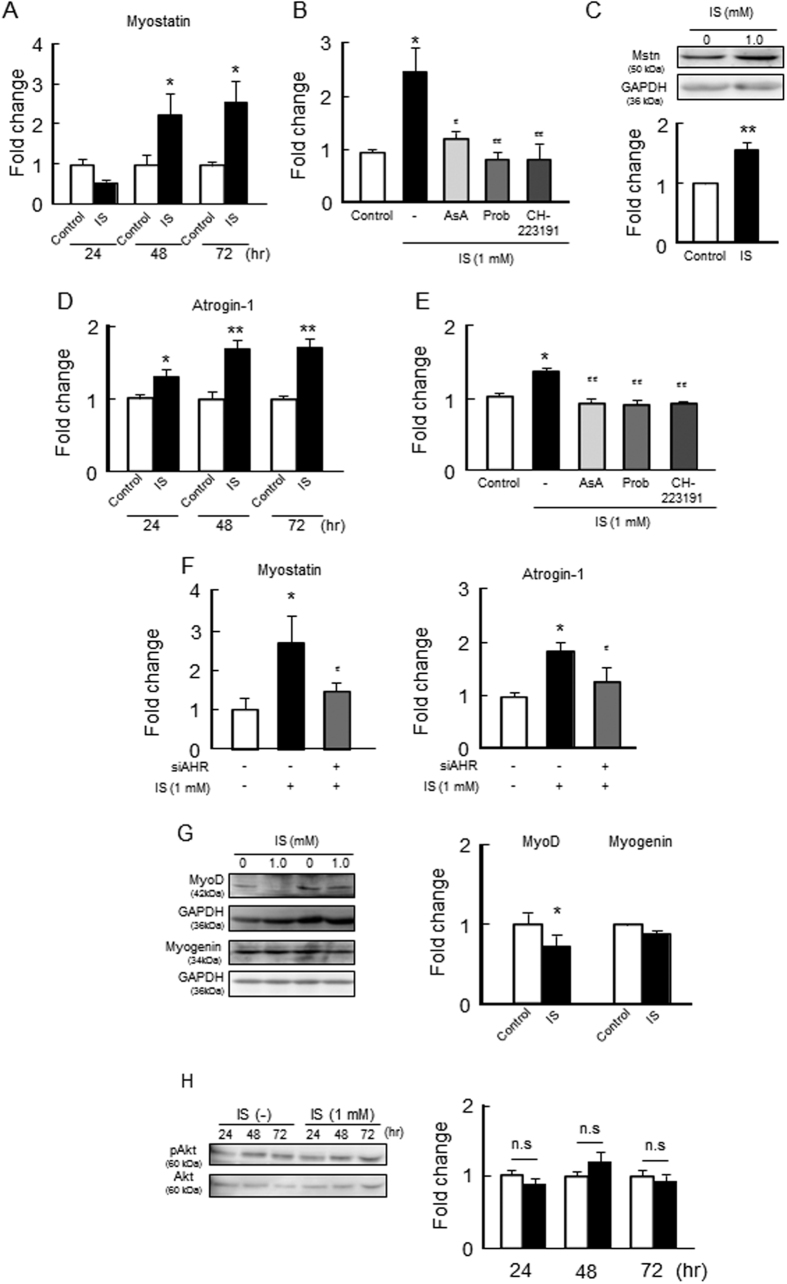
Effect of IS on the expression of myostatin, skeletal muscle atrophy- or myogenic-related genes and Akt phosphorylation in C2C12 myoblast cells. Effect of IS on (**A,B**) mRNA and (**C**) protein expression of myostatin, mRNA expression of (**D,E**) atrogin-1 were determined by real time RT-PCR. (**F**) Effect of AHR RNAi on IS-induced myostatin or atrogin-1 expression was determined by real time RT-PCR. (**G**) Protein expression of MyoD and myogenin in C2C12 myoblast cells were determined by Western blots. (**H**) Phosphorylation of Akt was detected by Western blots. C2C12 myoblast cells were starved with serum free medium for 2 hr, and then treated with IS for 24, 48 or 72 hr. After the incubation, total RNA or a whole cell lysate was collected and mRNA or protein expressions in the C2C12 myoblast cells were determined. To determine the effect of inhibitors, the cells were co-incubated in the presence or absence of ascorbic acid (AsA, a ROS scavenger), probenecid (Prob, Oat inhibitor) and CH-223191 (AHR inhibitor) for 72 hr, and the mRNA expression of (**B**) myostatin or (**E**) atrogin-1 in C2C12 myoblast cells were determined. Data are expressed the means ± SEM (n = 3~4). *p < 0.05, **p < 0.01 compared with control. ^#^*p* < 0.05, ^##^*p* < 0.01 compared with IS only.

We also examined the effect of IS on the expression of muscle atrophy-related genes in C2C12 myoblast cells. As shown in Fig. 4A, the expression of myostatin mRNA began to increase at 48 hr. The protein expression of myostatin was also increased in the case of a 72 hr incubation of IS (Fig. 4C). The presence of IS also significantly increased the mRNA level of atrogin-1, an ubiquitin E3 ligase from 24 hr to 72 hr after IS treatment (Fig. 4D). Similar to ROS and inflammatory cytokines, increases in these mRNA expressions were significantly inhibited by the presence of ascorbic acid, probenecid or CH-223191 (Fig. 4B,E). To prove that the effect of IS on muscle atrophy is due to the AHR pathway, we performed additional experiments using AHR-siRNA in C2C12 cells. As a result, the AHR-siRNA treatment suppressed IS-induced muscular atrogin-1 and the induction of myostatin, strongly suggesting that AHR activation by intracellular IS contributes to muscle atrophy (Fig. 4F). On the other hand, IS reduced the expression of MyoD protein but has no effect on myogenin expression in C2C12 myoblast cells (Fig. 4G). Since the Akt pathway plays important roles for skeletal muscle hypertrophy by increasing muscle protein synthesis and decreasing protein degradation^28^,^29^, we examined the effect of IS on Akt phosphorylation in C2C12 myoblast cells. As shown in Fig. 4H, the IS treatment did not affect Akt phosphorylation.

### Effect of IS on ROS production and the expression of inflammatory cytokines, myostatin and atrogin-1 in C2C12 myotubes

**Figure 5:**
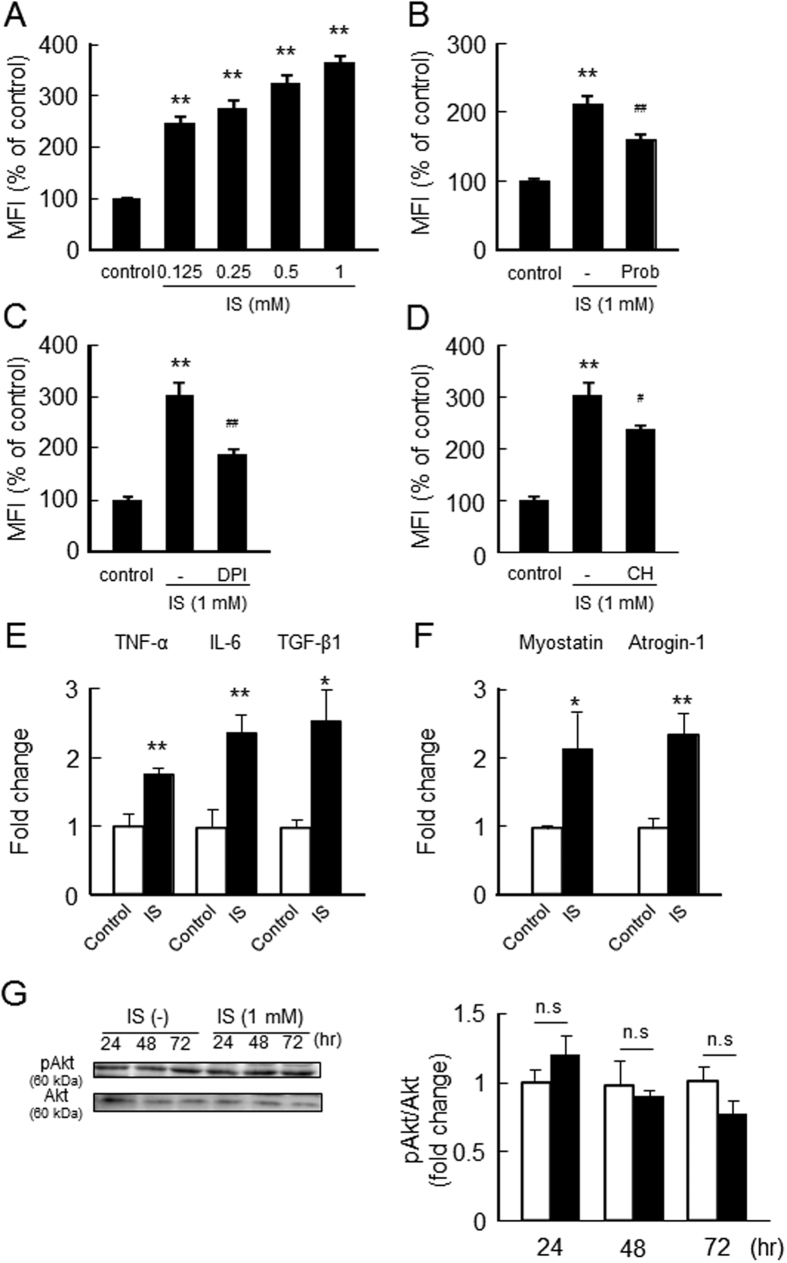
Effect of IS on the ROS production and expression of inflammatory cytokines, myostatin and atrogin-1 in C2C12 myotubes. C2C12 myotubes were differentiated for 4 days in differentiated medium. (**A**) To determine the ROS production, C2C12 myotubes were starved with serum free medium for 2 hr, and then treated with CM-H_2_DCFDA in D-PBS for 30 min. After removal of the D-PBS, the cells were treated with IS and incubated for 2 hr. Fluorescence intensity was measured at an excitation wavelength of 485 nm and an emission wavelength of 535 nm. To determine the effect of inhibitors of Oats, NADPH oxidase or AHR, C2C12 myotubes were incubated with CM-H_2_DCFDA for 30 min followed by incubation with (**B**) probenecid (Prob: 0.5 mM), (**C**) diphenylene iodonium (DPI: 50 μM), or (**D**) CH223191 (10 μM) for 30 min. The cells were then incubated with IS (1 mM) for 1 hr with an Oats inhibitor or 2 hr with others. C2C12 myotubes were starved with serum free medium for 2 hr, and then treated with IS for 72 hr. After incubation, total RNA was collected and the mRNA expression of (**E**) IL-6, TNF-α, TGF-β1, (**F**) myostatin and atrogin-1 were determined. (**G**) Akt phosphorylation was determined after 72 hr incubation with IS. Data are expressed the means ± SEM (n = 3~6). *p < 0.05, **p < 0.01 compared with control. ^#^*p* < 0.05, ^##^*p* < 0.01 compared with IS only.

To clarify whether IS acts on myotubes as well as myoblasts, we examined the effect of IS on ROS and inflammatory cytokines production in C2C12 myotubes. As shown in Fig. 5A, IS induced ROS production in C2C12 myotubes. This was significantly inhibited in the presence of probenecid, DPI and CH-223191 (Fig. 5B–D). IS also induced the expression of inflammatory cytokines (TNF-α, IL-6 and TGF-β1) (Fig. 5E), myostatin (Fig. 5F) and atrogin-1 (Fig. 5F) expression in C2C12 myotubes, while it had no effect on Akt phosphorylation (Fig. 5G). These *in vitro* data using C2C12 myoblast cells and myotubes suggest that IS potentiates the induction of skeletal muscle atrophy by inhibiting cell proliferation and myotube formation through increasing the expression of myostain and atrogin-1.

### Effect of IS administration on skeletal muscle mass in half-nephrectomized mice

To confirm the *in vitro* data, we further examined the issue of whether the administration of IS to half-nephrectomized (1/2 Nx) mice induced muscle atrophy. In another model such as the 5/6 nephrectomy model, not only IS but also other uremic toxin such as *p*-cresyl sulfate levels in plasma are also increased. Therefore, to clarify the contribution of IS, we used IS-overloaded 1/2 Nx mice. In this experiment, IS (100 mg/kg/day) was intraperitoneally administered to 1/2 Nx mice for a period of 12 weeks. Table 1 shows body weight, muscle weight and renal function of the 1/2 Nx (control) and IS-treated 1/2 Nx mice. At 12 weeks after the IS treatment, no significant differences of serum creatinine and BUN were observed between control and IS-treated 1/2 Nx mice. In addition, these values are comparable to those of healthy mice. However, the weight of body and muscle (tibialis anterior, soleus, gastrocnemius) in the IS-treated 1/2 Nx mice were significantly lower than those in control group (Table 1), suggesting that exogenous IS induces muscle atrophy in this model.

### Pharmacokinetic properties of exogenous IS in half-nephrectomized mice

**Figure 6:**
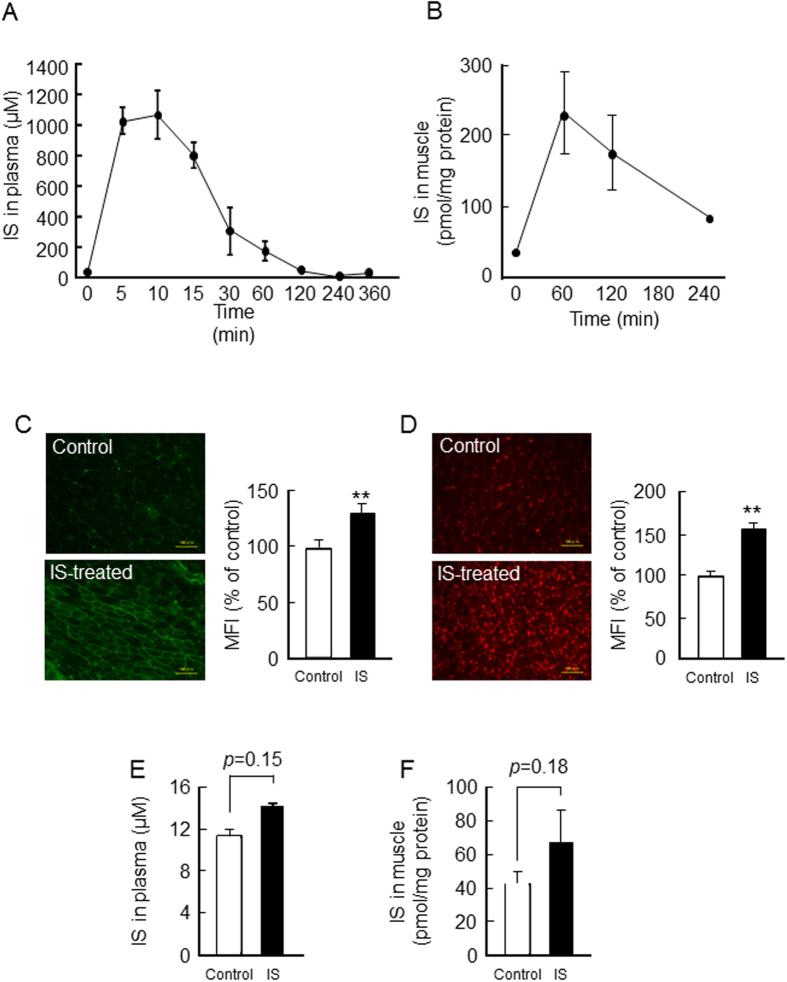
Plasma and skeletal muscle concentration of IS in IS-administered 1/2 Nx mice. (**A**) Plasma concentration-time profile of IS after intraperitoneal administration of IS (100 mg/kg) into 1/2 Nx mice was determined by HPLC methods. (**B**) The gastrocnemius concentration of IS was measured after IS administration. (**C**) Immunofluorostaining image of IS using anti-IS antibody on gastrocnemius was performed. (**D**) DHE staining of gastrocnemius was performed, and significant increasing of superoxide production was observed 1 hr after IS administration. (**E**) Plasma and (**F**) gastrocnemius muscle concentration of IS were measured at the trough level after 12 weeks IS administration. Scale bar = 100 μm. Data are expressed the means ± SEM (n = 3~8). **p < 0.01 compared with control.

So far, there is no information regarding the skeletal muscle distribution of IS *in vivo*. Therefore, we characterized the pharmacokinetic properties of IS in 1/2 Nx mice. As shown in Fig. 6A, the plasma concentration of IS reached a maximum at 10 min after an intraperitoneal administration and was rapidly eliminated from the blood within 4 hr. This could be due to the preservation of sufficient renal elimination in this model. However, the amounts of IS in skeletal muscle (gastrocnemius) which originates from the blood circulation was still sustained at 4 hr after the administration of IS (Fig. 6B). This was further confirmed by an immunostained image of IS using anti-IS antibody on a gastrocnemius section (Fig. 6C). At the same time, we also assessed ROS production in the skeletal muscle of IS treated 1/2 Nx mice using DHE, a fluorescent dye. As a result, marked fluorescence was clearly observed in the gastrocnemius section (Fig. 6D). Interestingly, the pattern of the immunostaining image of IS was similar to that of the DHE fluorescence, suggesting that IS induces superoxide production in skeletal muscle *in vivo*.

At 12 weeks after IS administration, plasma trough levels of IS tended to increase as the result of IS administration (14.3 ± 1.9 μM for IS-treated mice; 11.0 ± 1.3 μM for control, *p* = 0.15) (Fig. 6E). At the same time, IS level in gastrocnemius also tends to increase (67.1 ± 19.7 pmol/mg protein for IS-treated mice; 43.0 ± 7.0 pmol/mg protein for control, *p* = 0.18) (Fig. 6F).

### Effect of the chronic administration of IS on the expression of inflammatory cytokines, muscle atrophy-related or myogenic-related genes expression or Akt phosphorylation in the skeletal muscle of half-nephrectomized mice

**Figure 7:**
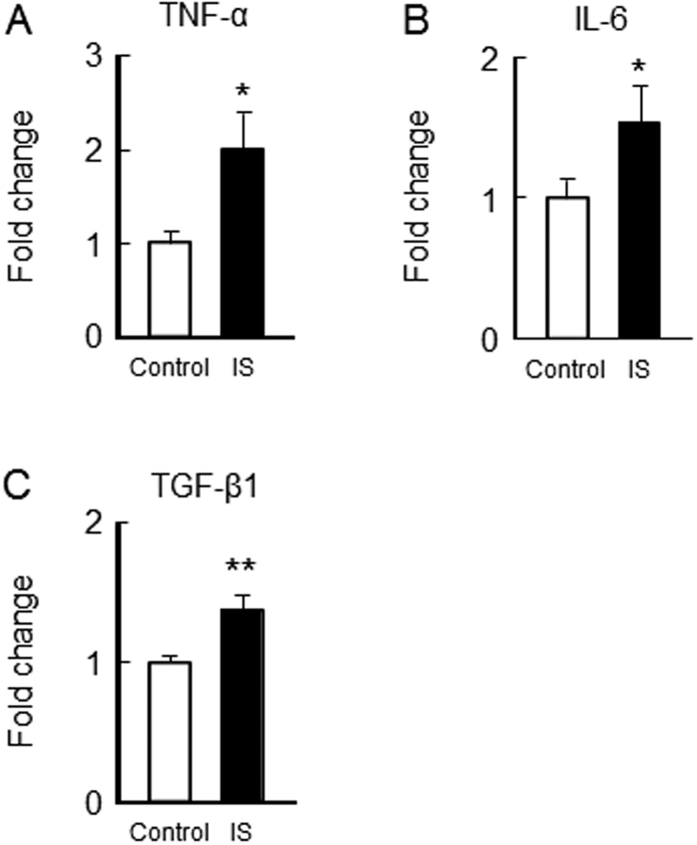
Effect of IS administration on inflammatory cytokines expression in the skeletal muscle of 1/2 Nx mice. After IS administration for 12 weeks, the mRNA expression of (**A**) TNF-α, (**B**) IL-6 and (**C**) TGF-β1 in gastrocnemius were determined by real time RT-PCR. Data are expressed the means ± SEM (n = 6~8). *p < 0.05, **p < 0.01 compared with control.

We examined the effect of chronic IS administration on the expression of TNF-α, IL-6 and TGF-β1 in gastrocnemius of 1/2 Nx mice. In the IS-treated group, TNF-α, IL-6 and TGF-β1 mRNA expression in the gastrocnemius were significantly increased compared with the control group (Fig. 7A–C).

**Figure 8:**
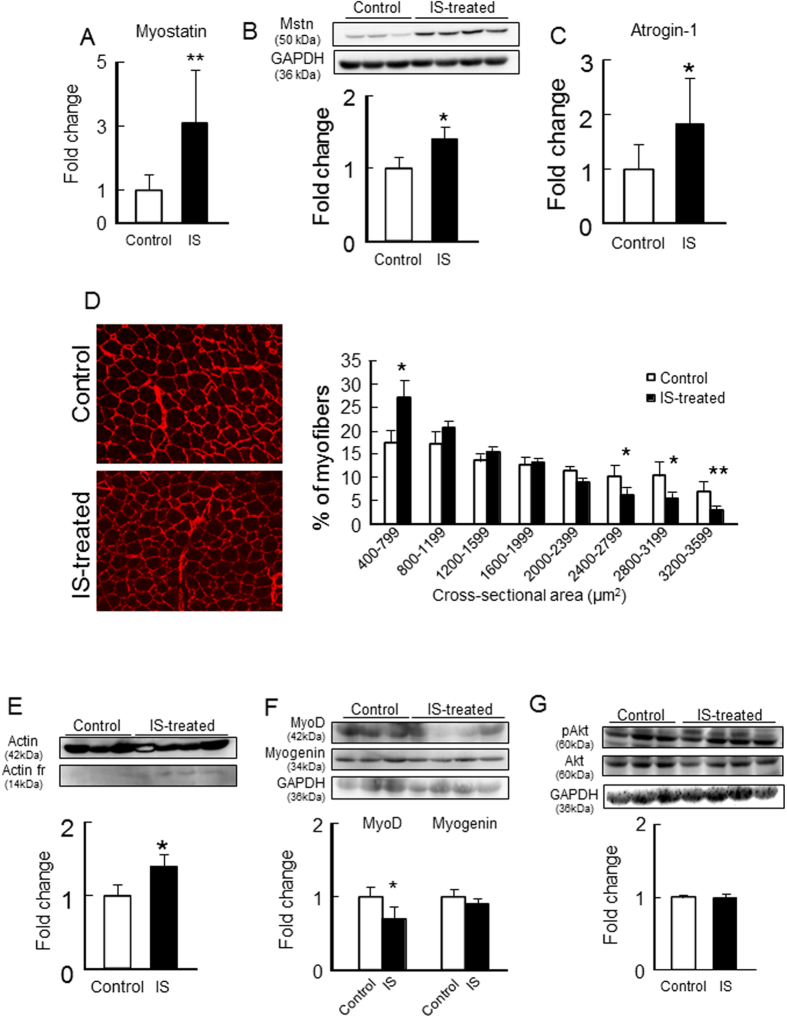
Effect of IS administration on myostatin expression and muscle atrophy- or myogenic-related genes expression or Akt phosphorylation in the skeletal muscle of 1/2 Nx mice. After IS administration for 12 weeks, (**A**) mRNA and (**B**) protein expressions of myostatin in gastrocnemius were determined by real time RT-PCR and Western blots. mRNA expression of (**C**) atrogin-1 in gastrocnemius were determined by real time RT-PCR. (**D**) Cryosections of tibialis anterior muscles were immunostained with anti-laminin to assess myofiber size. (**E**) Muscle degradation marker, 14 kDa actin fragment was determined by Western blot. (**F**) MyoD and myogenin expression in gastrocnemius were determined by Western blots. (**G**) Akt phosphorylation in gastrocnemius was determined by Western blots. Relative intensity of pAkt/Akt was quantified using the ImageJ software. Data are expressed the means ± SEM (n = 6~8). *p < 0.05, **p < 0.01 compared with control.

As shown in Fig. 8A,B, the chronic administration of IS resulted in a significant increase in the mRNA and protein expression of myostatin in the gastrocnemius. Similarly, IS also significantly increased the mRNA expression of atrogin-1 (Fig. 8C). The administration of IS resulted in a significant decrease in the average cross-sectional area of myofibers (Fig. 8D). In addition, direct muscle wasting evidence (14-kD actin fragment) was also detected in the IS administration group (Fig. 8E). In addition, IS reduced MyoD expression but had no effect on the expression of myogenin (Fig. 8F). However, IS did not affect the phosphorylation of Akt in the gastrocnemius (Fig. 8G). These data are entirely consistent with the results obtained from *in vitro* experiments using C2C12 cells, as discussed above.

## Discussion

Muscle atrophy is a well-established complication of CKD. During muscle wasting, abnormal levels of ROS and inflammatory cytokines are observed in skeletal muscle^15^,^30^. Therefore, it has been pointed out that a close relationship exists between oxidative stress, inflammation and muscle atrophy. However, the overall mechanism responsible for these effects are not fully understood, and in particular, there is little information regarding the contribution of uremic toxins. In this study, we found, for the first time, that among six uremic toxins, IS has the strongest ability to increase ROS production and to inhibit cell proliferation and differentiation in C2C12 cells. Moreover, *in vitro* and *in vivo* experiments showed that IS increased the expression of myostatin and atrogin-1, in addition to inflammatory cytokine (TNF-α, IL-6 and TGF-β1) expression in muscle, with the result that the administration of IS to 1/2 Nx mice caused a decrease in muscle weight (tibialis anterior, soleus and gastrocnemius) without affecting renal function. These findings show that IS is a potent contributor to the development of skeletal muscle atrophy associated with CKD.

There is strong evidence to indicate that myostatin acts as a key mediator of muscle atrophy in CKD due to the reduction of protein synthesis and an increase in protein degradation^9^,^10^,^29^. There is, however, no report on the effect of IS on muscle atrophy-related genes available as of writing this. Previously, Zhang *et al*. reported that myostatin expression in muscle was increased in an advanced CKD model which was created by loading a 40% protein diet to 5/6 nephrectomized mice^9^. In their study, increased ROS-induced TNF-α expression triggers myostatin production by a NF-κB dependent-pathway, which further stimulated the production and release of IL-6 in muscle. Sriram *et al*. also demonstrated that myostatin-induced TNF-α production *via* NF-κB signaling further increased ROS levels *via* the activation of NADPH oxidase. The induced ROS results in a feed forward loop that further increases myostatin expression *via* the NF-κB signaling of TNF-α^15^. Unfortunately, they did not identify the substances that induced both ROS and inflammatory cytokines in the CKD model mice. Previous studies demonstrated that IS induces both ROS and inflammatory cytokines through the activation of NADPH oxidase and the activation of NF-κB in kidney, bone, heart and blood vessels. Therefore, IS has a possibility to induce myostatin expression in skeletal muscle as well. Although Zhang’s group did not measure the plasma and tissue concentration of IS, an enhanced accumulation of IS in their CKD model is a possibility, because it has been reported that the intake of a high protein diet results in the enhancement of IS accumulation under CKD conditions.

In the present study, we found that after IS treatment, ROS was induced in C2C12 cells at 2 hr, and at 24 hr, mRNA expression of TNF-α then began to increase, and finally elevated levels of myostatin were observed at 48 hr (Figs 3 and 4). In addition, the mRNA of IL-6 was elevated at 72 hr after the IS treatment. By organizing in the order of these IS induced responses, it is possible that ROS derived from IS enhances TNF-α production in muscle cells, which in turn increases myostatin levels and further induces IL-6 production. Such a feed forward loop induced by IS is consistent with the results using advanced CKD model mice, as mentioned above.

In addition to myostatin, we also found that IS resulted in an increase in the expression of TGF-β1 and atrogin-1. Previously, Satori *et al*. reported that the activation of the TGF-β1 pathway induces muscle atrophy dependent on atrogin-1 *via* Smad2/3^31^. Moreover, Bonaldo *et al*. showed that the TGF-β1/Smad/atrogin-1 pathway plays a key role in proteolysis in muscle cells and myostatin also activates the Smad2/3 pathway^32^. These previous data provide comprehensive support for our data that IS induces proteolysis in muscle cells *via* multiple pathways including TGF-β1/Smad/atrogin-1 and myostatin/Smad/atrogin-1.

Myogenesis involves transcription factors including MyoD and myogenin. Our data show that IS reduces the protein expression of MyoD but had no effect on the expression of myogenin protein, under both *in vitro* and *in vivo* conditions. In addition, IS was found to have no effect on the mRNA expression of MyoD and myogenin (data not shown). A previous report demonstrated that atrogin-1 targets MyoD for its degradation through the ubiquitin proteasome-mediated system^33^. Myostatin inhibits myogenesis by lowering MyoD levels. Therefore, it is possible that IS could increase the degree of degradation of MyoD by inducing the formation of atrogin-1 or myostatin. In contrast, IS was found to have no effect on the expression of myogenin protein in our experimental systems. Although the reason for why IS did not affect the expression of myogenin protein is not clear at present, the issue of whether atrogin-1 or myostain increases or decreases myogenin expression remains a controversial point^34^,^35^.

In the present study, we found that the pro-oxidant properties of IS play an important role in the development of muscle atrophy. So far, several mechanisms have been proposed to explain IS induced tissue damage. Specifically, Enomoto and Niwa *et al*. showed that IS accumulates in the target cells *via* OAT (1, 3)^21^,^22^,^23^, and it exerts toxicity *via* the activation of cellular NADPH oxidase and AHR, which results in the overproduction of ROS and inflammatory cytokines. In the present *in vitro* study, we found a similar mechanism in which ROS derived from IS in C2C12 cells is significantly suppressed by the presence of inhibitors of Oat, NADPH oxidase and AHR. Moreover, the enhanced production of inflammatory cytokines, myostatin and atrogin-1 by IS was also inhibited by co-incubation with a ROS scavenger and an inhibitor of Oat and AHR. These findings strongly suggest the importance of the accumulation of IS in muscle cells, and IS-induced ROS production *via* the AHR/NADPH oxidase/NF-κB pathway triggers myostatin, atropgin-1, TNF-α and IL-6 production, which further leads to a muscle atrophy.

As of this writing, a number of AHR ligands have been identified and extensively investigated. It is generally believed that high-affinity AHR ligands must contain two or more cyclic or aromatic ring structures in order for them to be functional. Among such structures are polycyclic aromatic hydrocarbons, halogenated aromatic hydrocarbons and many naturally occurring flavonoids. Schroeder *et al*. first demonstrated that IS is a direct AHR ligand and leads to the activation of AHR^36^. In their study, the AHR-driven reporter activity of IS was found to be higher than that of IA. In addition, structure-function studies also revealed that the presence of a polycyclic aromatic ring and a sulfate group are important determinants for efficient AHR activation. No direct data have been reported to show that PCS, HA, KA and CMPF are not substrates for AHR. However, taking previous reports and their chemical structure information into consideration, it is unlikely that PCS, HA, KA and CMPF are substrates for AHR.

Our *in vitro* experiments (Figs 2~4) demonstrated that an AHR inhibitor significantly suppressed the IS-induced production of ROS, inflammatory cytokine expression and atrophy-related gene (atrogin-1 and myostatin) expression. In addition, AHR-siRNA also suppressed IS-induced atrogin-1 and myostatin expression. Taking these data into consideration, it appears that the AHR pathway is crucial for IS-induced muscle atrophy. To prove that the effect of IS on muscle atrophy is due to the muscular uptake of IS *via* the OAT and AHR pathway, future *in vivo* experiments using muscle specific OAT and AHR knockout mice could provide a better understanding of the role of OAT and AHR.

Akt controls both protein synthesis *via* mTOR, and protein degradation *via* transcription factors of the FoxO family^32^. So far, high levels of ROS or myostatin have been shown to inhibit Akt phosphorylation, leading to proteolysis in muscle^15^,^37^. Therefore, we expected that Akt phosphorylation in our model mice would be impaired. However, no change was observed in Akt phosphorylation, suggesting that IS unlikely affects Akt phosphorylation. Wang *et al*. demonstrated that the level of phosphorylated Akt was decreased by a 3 hr exposure of 1 mM IS in human kidney 2 cells (HK-2 cells)^38^. On the other hands, Sun *et al*. reported that IS- and PCS-overloaded 1/2 Nx mice showed increased levels of phosphorylated Akt in kidneys^39^. From these data, the effect of IS on the phosphorylation of Akt may be different in each experimental conditions and might well vary with the specific organ. Future studies will be needed on Akt phosphorylation. Based on the present limited data, we have no explanation for the reason for this phenomena. Similar experiments using other CKD model animals may be needed to clarify it.

We also examined the effects of water-soluble toxins such as oxalic acid and uric acid on ROS production in C2C12 myoblast cells. As a result, the uremic concentration of uric acid significantly increased intracellular ROS production but oxalic acid did not (data not shown). Therefore, uremic toxins other than protein-bound types may also be involved in muscle atrophy in CKD conditions. Further investigations will clealy be needed to verify this this point.

## Conclusion

**Figure 9:**
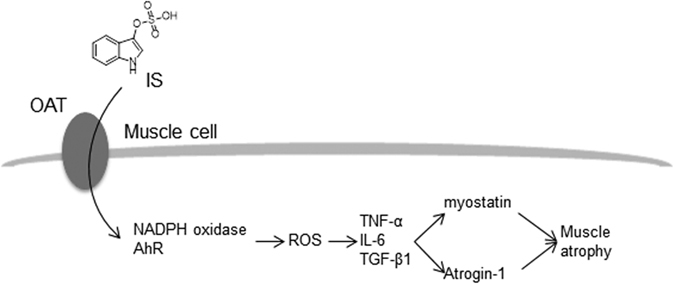
Proposed mechanism of IS-induced muscle atrophy. IS accumulates in muscle cells *via* Oat where IS activates NADPH oxidase and the AHR pathway to cause increased ROS production. The enhanced ROS production, in turn, triggers inflammatory cytokines (TNF-α, IL-6 and TGF-β1) induce myostatin and atrogin-1 expression that are involved in muscle wasting.

On the basis of the findings from *in vitro* and *in vivo* studies, we propose that the pro-oxidant property of IS induces skeletal muscle atrophy (Fig. 9). It appears that IS accumulates in muscle cells *via* Oat where IS activates NADPH oxidase and the AHR pathway to cause an increased ROS production. Enhanced ROS production, in turn, triggers the production of inflammatory cytokines (TNF-α, IL-6 and TGF-β1), and further induces the expression of myostatin and atrogin-1, which are involved in muscle wasting. These close relationships provide new insights into the mechanism by which the IS-induced loss of muscle mass in CKD, and could lead to new therapeutic strategies for treating muscle atrophy in patients with CKD.

## Materials and Methods

### Chemicals and materials

PCS was synthesized according to a previous report^40^ and the purity of product was confirmed by nuclear magnetic resonance spectra. IS, probenecid, diphenylene iodonium (DPI), dihydroethidium (DHE), CH-223191, rabbit polyclonal anti-mouse GAPDH antibody and anti-actin monoclonal antibody were purchased from Sigma-Aldrich (St Louis, MO). The rabbit anti-Oat1 polyclonal antibody (bs-0607R) and Rabbit anti-Oat3 polyclonal antibody (bs-0609R) were purchased from Bioss Inc (Boston, USA). Rabbit polyclonal anti-myostatin antibody was purchased from proteintech (Chicago, USA). The anti-myogenin monoclonal antibody, anti-myoD polyclonal antibody and anti-AHR monoclonal antibody were purchased from Santa Cruz Biotechnology (Texas, USA). The anti-myosin heavy chain antibody was purchased from R&D systems (Minesota, USA). Anti-IS antibody was kindly provided from Kureha Corporation (Tokyo, Japan). The antibiotic and antimycotic mixture (10000 U/ml penicillin, 10000 μg/ml streptomycin, 25 μg/ml amphotericin B), ascorbic acid was purchased from nacalai tesque (Kyoto, Japan). Dulbecco’s modified eagle medium (DMEM), dulbecco’s phosphate-buffered saline (D-PBS), 5-(and 6)-chloromethyl-2′,7′-dichlorodihydrofluorescein diacetate (CM-H_2_DCFDA) was purchased from Gibco (Invitrogen, Grand Island, NY). All methods were carried out in accordance with approved guidelines. All experimental protocols were approved by Kumamoto University.

### Cell culture

Mouse C2C12 myoblast cells were purchased from the RIKEN bioresource center cell bank (Ibaraki, Japan). C2C12 myoblast cells were cultured in DMEM supplemented with 10% fetal bovine serum (Hyclone Laboratories, Logan UT, USA), 100 U/ml penicillin, 100 μg/ml streptomycin and 0.25 μg/ml amphotericin B, and maintained under 37 °C and 5% CO_2_. After reached semi-confluence (80–90%) the medium was changed to differentiation medium (DMEM supplemented with 2% heat inactivated horse serum (Sigma-Aldrich)) to induce differentiation (myotube formation).

### Cell proliferation assay

C2C12 myoblast cells were seeded at 96-wellplate and cultured overnight. After cell adhesion, cultured medium was changed containing with uremic toxins (IS, IA, PCS, HA, KA, CMPF) and cultured for 72 hr. Adherent cells were fixed with fixative (10% formaldehyde (nacalai tesque, Kyoto Japan) and 0.9% saline). Adherent cells were assayed by a methylene blue assay, as described previously^41^.

### Cell differentiation and tubular formation assay

C2C12 myoblast cells were seeded at 12-well plate and cultured overnight. After cell adhesion, cultured medium was changed differentiation medium containing with uremic toxins (IS, IA, PCS, HA, KA, CMPF) and cultured for 7 days. The cell density in each treatment group was the same before changing the culture medium to differentiation medium. Cell morphology was observed by microscopy (Keyence, BZ-8000 microscope, Osaka, Japan). Average myotube diameters of at least 50 myotubes were determined to be 40 μm along the length of the myotube.

### ROS production assay

For the ROS production assay, CM-H_2_DCFDA, a ROS sensitive fluorescent dye, was used as the ROS detection probe. C2C12 myoblast cells were seeded in a 96-well plate and cultured overnight. After cell adhesion, cells were washed with PBS and starved with serum free medium for 2 hr, and then treated with CM-H_2_DCFDA (2.5 μM) for 30 min in D-PBS. After removing the D-PBS, the cells were incubated with uremic toxins (IS, IA, PCS, HA, KA, CMPF) in D-PBS for 2 hr. To determine the effect of inhibitors of Oats, NADPH oxidase or AHR on the IS-induced ROS production, cells were incubated with CM-H_2_DCFDA for 30 min in D-PBS. After removing the D-PBS, the cells were incubated with Oats inhibitors (probenecid (0.5 mM)), NADPH oxidase inhibitor (diphenylene iodonium: DPI (50 μM)) or AHR inhibitor (CH223191 (10 μM)) for 30 min, and then incubated with IS (1 mM) in the presence or absence of Oats inhibitors (1 hr) and others (2 hr). Fluorescence intensity was measured at an excitation wavelength of 485 nm and an emission wavelength of 535 nm using a fluorescence microplate reader SPECTRAfluor Plus (Tecan, Ma¨nnedorf, Switzerland).

### Animal experiments

All animal experiments were carried out in accordance with approved the guidelines of Kumamoto University for the care and use of laboratory animals. All animal experiments and procedures were approved by Kumamoto University. C57BL/6JJmsSlc mice (9 weeks, male) were purchased from Japan SLC, Inc (Shizuoka, Japan), and housed on a 12 hr day/night cycle. Half nephrectomized model mice were produced as described in previous reports^39^,^42^. Mice were anesthetized with pentobarbital (50 mg/kg, ip) and the right kidney was removed. At 1 week after surgery, mice were administrated with IS (100 mg/kg/day, ip) for 12 weeks. The control mice were administrated with PBS for 12 weeks at same volume. At the end of the study, mice were anesthetized with diethyl ether and blood, kidney, gastrocnemius, tibialis anterior and soleus were collected.

### HPLC analysis

The concentration of IS in plasma and skeletal muscle tissue were measured by an HPLC method, as described previously^43^. In brief, plasma or tissue (gastrocnemius) homogenate extracted with RIPA buffer containing 150 mM NaCl, 1% nonidet P-40, 10 mM Tris-HCl (pH7.4), 1% protease inhibitor cocktail (nacalai tesque, Kyoto, Japan), was mixed with acetonitrile (1:9, v/v for plasma sample or 1:3, v/v for the tissue homogenate sample) and centrifuged at 12000 × g for 10 min. The supernatant was then collected and mixed with ultrapure water (1:1, v/v for a plasma sample or 3:2, v/v for a tissue homogenate sample). The sample was loaded to HPLC with 20 μl for plasma sample and 50 μl for tissue homogenate. The HPLC system consisted of an Agilent 1100 series intelligent pump and a fluorescence spectrophotometer. A LiChro-sorb RP-18 column (Cica Merk, Tokyo, Japan) was used as the stationary phase. The mobile phase consisted of 0.2 M acetate buffer (pH4.0)-acetonitrile (3:1, v/v) for IS. The flow rate was 1.0 ml/min. IS was detected by means of a fluorescence monitor with excitation/emission wavelengths set to 280 and 375 nm, respectively.

### Western blotting analysis

Total protein was extracted by using RIPA buffer containing 150 mM NaCl, 1% nonidet P-40, 10 mM Tris-HCl (pH7.4), 1% protease inhibitor cocktail and 1% phosphatase inhibitor cocktail (nacalai tesque, Kyoto, Japan). A 30 μg sample of protein was mixed with sample buffer containing 50 mM dithiothreitol was boiled at 100 °C and separated with 10% sodium dodecyl sulfate-poly-acrylamide gel electrophoresis. Proteins were transferred polyvinylidene fluoride membrane, and then immunoblotted with antibodies against mouse Oat1, Oat3, myostatin, Akt, p-Akt, MyoD, myogenin, GAPDH, α-actin, myosin heavy chain and AHR under the room temperature for 1 hr. The sample was then immunoblotted with horseradish peroxidase conjugated secondary antibody at room temperature for 1 hr. The intensity of each band was detected using LAS4000mini (GE Healthcare, UK Ltd, Backinghamshire, England) and quantified using ImageJ software. The densitometric intensity was normalized with GAPDH expression.

### Real time RT-PCR analysis

Total RNA was extracted using RNAiso PLUS (TaKaRa Bio Inc., Shiga, Japan) according to the manufactures protocol. The concentration and the purity of the RNA extract was determined by measuring the absorbance at 260 nm and 280 nm. The cDNA was synthesized using PrimeScript^®^ RT master mix (Perfect Real Time) (TaKaRa Bio Inc.). Quantitative real time RT-PCR analysis of IL-6, TNF-α, TGF-β1, myostatin, atrogin-1, MyoD, myogenin and GAPDH was performed in an iCycler thermal cycler (Bio-Rad) with an iQ5 qRT-PCR detection system attached (Bio-Rad) using SYBR^®^ Premix Ex TaqII (Perfect Real Time) (TaKaRa Bio Inc.). PCR amplifications were performed under the following conditions: 95 °C for 3 min, for 40 cycles at 95 °C for 10 s (denaturation step), at 60 °C for 1 min (annealing/extension steps). The primers used are shown in supplemental Table 1. The threshold cycle (Ct) values for each gene amplification were normalized by subtracting the Ct value calculated for GAPDH.

### Small interfering RNA experiments

AHR Stealth RNAi or Stealth RNAi negative control duplex was transfected in C2C12 myoblast cells, according to the manufacturer’s recommendations. Cells grown in 12 well plates were transfected with 10 nM of siRNA/well using Lipofectamine RNAiMAX (Invitrogen). After a 72 hr period of incubation, the cells were used for the experiments.

### Immunostaining of tissue sections

4 μm-thick of frozen sections of skeletal muscle were washed with PBS, followed by blocking with Block Ace (DS Pharma Biomedical Co., Ltd, Osaka, Japan) at room temperature for 30 min. The anti-IS antibody or anti-laminin antibody (Sigma-Aldrich) was then reacted for 90 min at room temperature. After the reaction, the slides were washed and reacted with the secondary antibody (Alexa Fluor 488 goat anti-mouse IgG (H + L), Alexa Fluor 564 goat anti-rabbit IgG (H + L), Invitrogen, Eugene, USA) for 90 min, followed by observed by microscopy (Keyence, BZ-8000 microscope, Osaka, Japan). Fluorescence of the skeletal muscle sections was quantified using ImageJ software (Bethesda, MD). The mean fluorescence intensity was quantified.

### Dihydroethidium staining

The amounts of superoxide production in skeletal muscle was determined using dihydroethidium (DHE), a superoxide detection probe, following a previous protocol^20^. 10 μm-thick of frozen section of skeletal muscle was reacted with DHE (5 μM) at 37 °C for 30 min, and then observed by microscopy. DHE fluorescence of the skeletal muscle sections was quantified using the ImageJ software (Bethesda, MD). The mean fluorescence intensity was quantified.

### Statistical analyses

The means for two group data were compared by the unpaired t-test. The means for groups were compared by analysis of variance followed by Tukey’s multiple comparison. A probability value of *P* < 0.05 was considered to be significant.

## Additional Information

**How to cite this article**: Enoki, Y. *et al*. Indoxyl sulfate potentiates skeletal muscle atrophy by inducing the oxidative stress-mediated expression of myostatin and atrogin-1. *Sci. Rep.* **6**, 32084; doi: 10.1038/srep32084 (2016).

## Supplementary Material

Supplementary Information

## Figures and Tables

**Table 1:** Body weight, muscle weight and renal function profile for 1/2 nephrectomized (1/2 Nx: control) and IS-treated 1/2 Nx mice.

	Control (1/2 Nx mice)	IS-treated 1/2 Nx mice	*P* value
Initial body weight (g) (at 0 week)	24.0 ± 0.6	23.1 ± 0.3	0.09
Final body weight (g) (at 12 weeks)	28.3 ± 0.6	25.3 ± 0.6	*0.01
BUN (mg/dl)	29.6 ± 1.4	28.0 ± 1.9	0.27
Creatinine (mg/dl)	0.30 ± 0.03	0.24 ± 0.04	0.13
Tibialis anterior (mg)	59.3 ± 1.9	54.1 ± 2.0	*0.045
Soleus (mg)	11.0 ± 0.5	8.9 ± 0.1	**0.0002
Gastrocnemius (mg)	174.7 ± 2.6	157.7 ± 2.7	**0.0003

Data are expressed as the mean ± SEM. *p < 0.05, **p < 0.01 compared with 1/2 Nx mice.
