# Supplementary material for: Indoxyl sulfate potentiates skeletal muscle atrophy by inducing the oxidative stress-mediated expression of myostatin and atrogin-1
Source: Sci Rep. 2016 Aug 23;6:32084. doi: 10.1038/srep32084 (PMC4994088; doi:10.1038/srep32084)
Supplement: Supplementary Information [file srep32084-s1.docx]

**Indoxyl sulfate potentiates skeletal muscle atrophy by inducing the oxidative stress-mediated expression of myostatin and atrogin-1**

Yuki Enoki^1^, Hiroshi Watanabe^1,2^, Riho Arake^1^, Ryusei Sugimoto^1^, Tadashi Imafuku^1^, Yuna Tominaga^1^, Yu Ishima^1,2^, Shunsuke Kotani^3^, Makoto Nakajima^3^, Motoko Tanaka^4^, Kazutaka Matsushita^4^, Masafumi Fukagawa^5^, Masaki Otagiri^6,7^, and Toru Maruyama^1,2^

**Table S1.** Primers in real time RT-PCR

| Target gene | Forward | Reverse | bp |
| --- | --- | --- | --- |
| IL-6 | 5’-TCTCTGCAAGAGACTTCCATCC-3’ | 5’-AGACAGGTCTGTTGGGAGTG-3’ | 126 |
| TNF-α | 5’-CATGAGCACAGAAAGCATGATCCG-3’ | 5’-AAGCAGGAATGAGAAGAGGCTGAG-3’ | 121 |
| TGF-β | 5’-TACCATGCCAACTTCTGTCTGGGA-3’ | 5’-TGTGTTGGTTGTAGAGGGCAAGGA-3’ | 91 |
| Myostatin | 5’-CTGTAACCTTCCCAGGACCA-3’ | 5’-TCTTTTGGGTGCGATAATCC-3’ | 197 |
| Atrogin-1 | 5’-CAGAGAGGCAGATTCGCAAG-3’ | 5’-GGTGACCCCATACTGCTCTC-3’ | 116 |
| MyoD | 5’-TGGCATGATGGATTACAGCG-3’ | 5’-GAGATGCGCTCCACTATGCT-3’ | 156 |
| Myogenin | 5’-TCCCAACCCAGGAGATCATT-3’ | 5’-TCAGTTGGGCATGGTTTCGT-3’ | 136 |
| GAPDH | 5’-AACTTTGGCATTGTGGAAGG-3’ | 5’-ACACATTGGGGGTAGGAACA-3’ | 223 |
